# Personality and Healthy Sleep: The Importance of Conscientiousness and Neuroticism

**DOI:** 10.1371/journal.pone.0090628

**Published:** 2014-03-20

**Authors:** Katherine A. Duggan, Howard S. Friedman, Elizabeth A. McDevitt, Sara C. Mednick

**Affiliations:** Department of Psychology, University of California Riverside, Riverside, California, United States of America; Hospital General Dr. Manuel Gea González, Mexico

## Abstract

Although previous research has shown personality and sleep are each substantial predictors of health throughout the lifespan, little is known about links between personality and healthy sleep patterns. This study examined Big Five personality traits and a range of factors related to sleep health in 436 university students (*M*_age_ = 19.88, *SD* = 1.50, 50% Male). Valid self-report measures of personality, chronotype, sleep hygiene, sleep quality, and sleepiness were analyzed. To remove multicollinearity between personality factors, each sleep domain was regressed on relevant demographic and principal component-derived personality factors in multiple linear regressions. Results showed that low conscientiousness and high neuroticism were the best predictors of poor sleep (poor sleep hygiene, low sleep quality, and increased sleepiness), consistent with other research on predictors of poor health and mortality risk. In this first comprehensive study of the topic, the findings suggest that personality has a significant association with sleep health, and researchers could profitably examine both personality and sleep in models of health and well-being.

## Introduction

There is a rich tradition of research that links personality with substantial health outcomes (e.g., [1], [2]). Using the Big Five, which is an integrative framework that describes regularities in behavior and hierarchically organizes them into broad personality traits [3], both high conscientiousness and low neuroticism have been linked with better health outcomes. Conscientiousness, which describes socially-prescribed impulse control, task- and goal-oriented behavior, planfulness, persistence, and dependability [4], [5], has been associated with decreased mortality risk in clinical, elderly, and healthy populations [6], as well as in individuals followed since childhood [7]. People high in conscientiousness live longer lives because they engage in more health-promoting behaviors, including more physical activity, healthier diets, lower substance use, and fewer risky behaviors [8], and because they have more stable relationships and better integration into their communities [9]. Neuroticism, which describes individuals who are emotionally reactive and tend to experience more negative emotions such as anxiety, hostility, nervousness, and depression [4], is associated with health risk. People high in neuroticism are at greater risk of poor mental and physical health [10] and increased mortality [11]. Most people high in neuroticism have fewer health-promoting behaviors and engage in more risk taking [12], and they are also very sensitive and more likely to report somatic complaints [13], possibly relevant to poor sleep. However, some neurotic individuals—high in prudent worrying—are not at increased disease risk [14].

Multiple aspects of sleep predict substantial health outcomes. Sleep duration is the most commonly investigated domain with respect to health and has been associated with obesity [15], diabetes and insulin resistance [16], and cardiovascular disease [17]. Longitudinally, meta-analyses have confirmed that both short and long sleep durations are associated with increased mortality risk [18], [19]. However, other aspects of sleep are also important to health and well-being, including habitual behaviors (e.g., chronotype and sleep hygiene) and subjective experience (e.g., sleep quality and sleepiness). Chronotype (the preference for activity in the morning or evening) and sleep hygiene (health-promoting behaviors related to sleep) represent habitual aspects of sleep behavior that may lead to physical health benefits. For example, morning people engage in more health-promoting behaviors [20] and interventions that improve sleep hygiene reduce migraine frequency in children and adolescents [21]. On the other hand, sleep quality (composed of self-reported quantitative aspects of sleep such as sleep duration as well as qualitative aspects, including the “depth” and “restfulness” of sleep [22]) and sleepiness represent more subjective self-reports of sleep health. Poor sleep quality has been associated with increased somatic complaints and poor mental health [23], [24] as well as increased mortality risk [25], [26]. Daytime sleepiness, which may be the product of poor sleep quality, is also associated with lower general health perceptions, energy levels, well-being, and functional status [27].

Although personality and sleep each have been associated with health, surprisingly little is known about the relationship between personality and sleep. Most research has focused on personality and chronotype, reporting that conscientious people tend to be morning types whereas neurotics tend to be evening types [28], [29]. Conscientiousness has been associated with better sleep quality [30], [31]. Neuroticism has also been linked with poor sleep quality [30], higher daytime sleepiness, and worse sleep hygiene [32]. However, much of the previous research used clinical samples, limited analyses to some indicators of personality or sleep, or did not use valid, reliable measures of both personality and sleep. Thus, prior studies have not conducted a comprehensive examination of sleep and personality. If sleep and personality have stable associations, then there would be substantial implications for causal models of sleep and health. The present study is the first to examine links between Big Five categories of conscientiousness, neuroticism, agreeableness, extraversion, and openness with chronotype, sleep hygiene, sleep quality, and sleepiness in a diverse college student sample, using well-validated measures.

## Methods

### Measures

436 university students (*M*age = 19.88, *SD* = 1.50; 50% Male) gave informed consent to participate in a research study for course credit. Participants were brought into the lab to complete the survey. Although consent and survey responses were documented anonymously via computer, a researcher gave clear instructions on how to complete the survey and was available to answer questions. Online data collection is an appropriate way to collect “high quality data inexpensively and rapidly” and yields data that are at least as reliable as traditional paper-and-pencil methods (e.g., [33]). After the participant read through the written consent form, consent was documented by the data collection system after participants clicked “next,” which indicated their consent to participate. This consent process and all research procedures were reviewed and approved by the University of California, Riverside Human Research Review Board.

Participants were ethnically diverse and most were of second-generation immigrant status (18% first generation, 68% second generation, 14% third generation or higher). Perceived socioeconomic status (SES) was assessed using a question that asked participants to rate their status from a 1 (low) to 10 (high) on a modified ladder scale similar to Adler, Epel, Castellazzo, and Ickovics [34]. As Adler et al. and others document, this may be a better predictor of health than monetary measures of SES, and such ladder-based SES questions are significantly related to indicators of objective SES, including income and educational degree [34]. Most participants were of mid-range SES (*M* = 6.74, *SD* = 1.85, *N* = 393; Note that there is a reduced *N* for the perceived SES analyses since the question was added mid-study). This is a relatively healthy sample (self-reported SF-36 General Health subscale, *N* = 434, *M* = 70.55, median = 70; see Table 1;[35]). Personality was assessed using the 44-item Big Five Inventory [36], which has high test-retest reliability (*r*_avg_ = .84) and maps well with peer reports of personality (*r*_avg_ = .56; [37]). Means in our sample were comparable to previous research using the Big Five in North American college students (e.g., [33]). Items were answered using a 5-point rating scale in which a 1 meant “disagree strongly” and a 5 meant “agree strongly.” Higher scores on each factor indicate higher levels of each personality trait. Sample items include “Makes plans and follows through with them” (conscientiousness); “Can be moody” (neuroticism); “Is generally trusting” (agreeableness); “Is full of energy” (extraversion); and “Is curious about many different things” (openness). See Table 1 for complete demographic and personality descriptives.

**Table 1:** Descriptive Statistics: Demographics and Personality.

Variable	*M* (*SD, N*) or Frequencies	Range (this sample)	Range (original scale)
*Demographics and Health*			
Age	19.88 (1.50, 436)	[18, 36]	----
Gender	218 males (50%); 218 females (50%)	----	----
Generation Status	80 1^st^ generation (18%); 296 2^nd^ generation (68%); 59 3^rd^ generation or higher (14%)	----	----
Subjective SES	6.74 (1.85, 393)	[1, 10]	[1, 10]
Self-Reported SF-36 General Health Subscale	70.55 (19.04, 434)	[10, 100]	[0, 100]
*Big Five Personality Traits*			
Conscientiousness	3.52 (0.59, 432)	[1.89, 5]	[1, 5]
Neuroticism	2.79 (0.76, 433)	[1, 4.63]	[1, 5 ]
Agreeableness	3.90 (0.57, 435)	[1.44, 5]	[1, 5 ]
Extraversion	3.25 (0.80, 435)	[1.38, 5]	[1, 5 ]
Openness	3.54 (0.56, 433)	[1.8, 4.9]	[1, 5 ]

**Figure 1:**
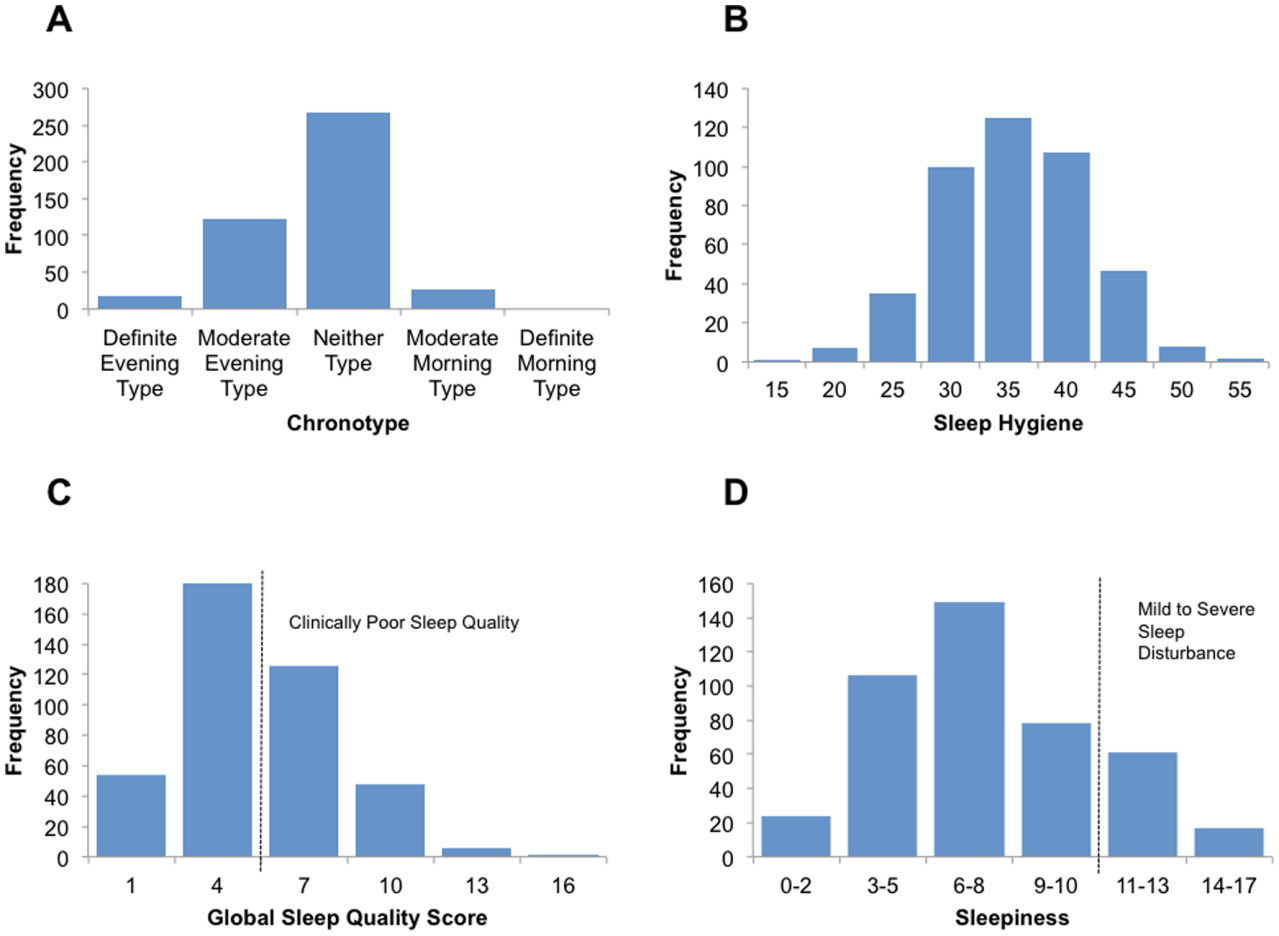
Distribution of Sleep Characteristics.

Chronotype, the relatively stable preference for activity in the morning or evening, was assessed using the Horne-Östberg Morningness-Eveningness Questionnaire (MEQ; [39]). The MEQ has high internal consistency reliability (α = 0.86) and high test-retest reliability (*r* = .89; [40]). The MEQ is related to peak body temperature, and self-reported bed/wake times in college students [35]. Items are answered either by indicating a time preference or making ratings on a 4-point scale. Higher scores on the MEQ indicate morningness, whereas lower scores indicate eveningness. Sample items include “Considering only your own ‘feeling best’ rhythm, at what time would you get up if you were entirely free to plan your day?” (participants answer by indicating the time) and “One hears about ‘morning’ and ‘evening’ types of people. Which ONE of these types do you consider yourself to be?” (rated using a 4-point scale). Most of the sample were neither types or moderate evening types (see Figure 1), and college samples typically tend toward eveningness [41]. For complete sleep descriptives, see Table 2.

**Table 2:** Descriptive Statistics: Sleep Variables.

Variable	Higher scores indicate…	*M* (*SD*, *N*)	Range (this sample)	Range (full scale)	Interpretation of Cut-points
Chronotype	more morningness	45.48 (8.51, 434)	[24, 69]	[16, 86]	definite morning type 70–86 (0%); moderate morning type 59–69 (6%); neither 42–58 (62%); moderate evening type 31–41 (28%); definite evening type 16–30 (4%)
Sleep Hygiene	worse sleep hygiene	35.46 (6.25, 432)	[17, 53 ]	[13, 78]	N/A
Sleep Quality	worse sleep quality	5.20 (2.69, 433)	[0, 15]	[0, 21]	scores >5 indicate clinically poor sleep quality; 42% of sample
Sleepiness	more daytime sleepiness	7.49 (3.28, 435)	[0, 17]	[0, 24]	scores >10 indicate a mild to severe sleep disturbance; 18% of sample

Sleep hygiene, practicing behaviors that facilitate sleep and avoiding behaviors that interfere with sleep, was assessed using the Sleep Hygiene Index [42]. The Sleep Hygiene Index has acceptable internal consistency reliability (α = 0.66) and test-retest reliability (*r* = .71) and is positively correlated with associated features of inadequate sleep hygiene (e.g., worrying about sleep; [37]). Items are rated using a 5-point rating scale, with a 1 meaning “never” and a 5 meaning “always.” Higher scores on the sleep hygiene index indicate worse sleep hygiene. Sample items include “I go to bed at different times from day to day” and “I use alcohol, tobacco, or caffeine within 4 h of going to bed or after going to bed.” Our sample mean of approximately 36 is close to the sample mean reported when the survey was validated, and suggests that most of our participants had average sleep hygiene practices. See Figure 1 for the distribution of sleep hygiene.

Sleep quality was measured using the Pittsburgh Sleep Quality Index (PSQI; [22]). This scale produces a global sleep quality score, which is created by summing each of the subscales: subjective sleep quality, sleep latency, sleep duration, habitual sleep efficiency, sleep disturbance, use of sleeping medication, and daytime dysfunction. The PSQI has high internal consistency (α = .83) and is sensitive and specific enough to discriminate healthy patients free of sleep complaints from patients with depression, disorders of maintaining sleep, disorders of initiating sleep, and disorders of excessive somnolence [22]. Items are answered either using a 4-point rating scale or by indicating time. Sample items include “During the last month, how often have you had trouble sleeping because you wake up in the middle of the night or early morning?” and “During the past month, how would you rate your sleep quality overall?” Higher scores on the PSQI indicate worse sleep quality, with scores greater than 5 indicating clinically poor sleep quality. When categorizing our participants this way, approximately 42% of them had clinically poor sleep quality, indicating that they exhibited severe problems in at least 2 sleep quality domains, or moderate problems in at least 3 sleep quality domains (see Table 2). See Figure 1 for the distribution of global sleep quality scores.

Trait daytime sleepiness was assessed using the Epworth Sleepiness Scale [43]. The Epworth Sleepiness Scale has high internal consistency (α = .73–.88) and high test-retest reliability in situations where sleepiness is expected to remain constant (*r* = .82), but scores do decrease when patients are treated for sleep disturbance (*t* = −9.59, *p*<.01; [44]). Additionally, it reliably distinguishes between patients without sleep complaints and patients with obstructive sleep apnea, narcolepsy, and idiopathic hypersomnia [38]. The scale asks participants “How likely are you to doze off or fall asleep in the following situations, in contrast to feeling just tired?” and sample situations include “sitting and reading” and “lying down to rest in the afternoon when circumstances permit.” Items are answered using a 4-point rating scale, with a 0 meaning “would *never* doze” and a 3 meaning “*high* chance of dozing.” Higher scores on the Epworth Sleepiness Scale indicate higher trait sleepiness, and scores above 10 indicate a mild to severe sleep disturbance may be contributing to daytime sleepiness. When categorizing our sample this way, approximately 18% of our participants are clinically sleepy throughout the day. See Figure 1 for the distribution of sleepiness.

### Analyses

Pearson correlations were used to examine associations among personality, demographic variables, and sleep characteristics. Because the Big Five personality traits have substantial naturally-occurring multicollinearity (Table 3), bivariate correlations are difficult to interpret in an unambiguous fashion (important in the current study; [45]). We therefore used a principal components analysis (PCA; [45]) to remove all shared variance between the personality traits by creating orthogonal personality factors using SAS® software, version 9.3 (SAS Institute, Cary, NC). Although the original Big Five personality measures were substantially intercorrelated (Table 3), the resulting PCA-derived personality factors are orthogonal and uncorrelated with each other. Furthermore, each factor has substantial (>.96) correlations with only one personality trait (correlations of each factor with the other traits are no larger than *r* = ±.17). Thus, PCA removed the substantial multicollinearity between the personality traits, and each factor represents only one personality trait. Then, after examining demographic predictors of sleep that were significant at the bivariate level in a regression framework (Model I), Model II examined the impact of the mean-centered PCA-derived personality components on each sleep characteristic. Regressing sleep on the PCA-derived personality factors obtains an identical *R* ^2^ to regressing sleep on the original personality factors but aids in interpretation because it removes shared variance between the personality variables [45]. Because all models were significant, and we were more concerned with effect sizes related to personality rather incremental increases in overall significance levels, the models presented here include the effects of all PCA-derived personality components to provide a full picture of the independent contributions of each trait (dropping predictors would not change parameter estimates for the PCA-derived personality components, but would trivially improve the overall significance of the models [45]). PCA-derived personality traits, age, and SES were mean-centered to aid in interpretability of parameter estimates. The reference category for gender is male and the reference category for generation status is second generation. Betas are interpreted as the unit change in sleep variable per 1-unit increase in the predictor variable at the mean of that predictor variable, controlling for the other variables in the model. Standardized betas are presented when appropriate to aid in interpretation of the models and are interpretable as the standard deviation change in the sleep variable per 1-standard deviation increase in the predictor variable, controlling for the other variables in the model.

**Table 3:** Correlations Between Big Five Personality Traits.

Variable	Neuroticism	Agreeableness	Extraversion	Openess
Conscientiousness	*r* = −.32	*r* = .37	*r* = .29	*r* = .24
	*p*<.001	*p*<.001	*p*<.001	*p*<.001
	*N* = 430	*N* = 432	*N* = 432	*N* = 430
Neuroticism	----	*r* = −.26	*r* = −.35	*r* = −.16
		*p*<.001	*p*<.001	*p* = .001
		*N* = 433	*N* = 433	*N* = 432
Agreeableness		----	*r* = .15	*r* = .17
			*p* = .002	*p*<.001
			*N* = 435	*N* = 433
Extraversion			----	*r* = .27
				*p*<.001
				*N* = 433

## Results

### Chronotype

High conscientiousness, (*r* = .35, *p*<.001), low neuroticism (*r* = −.18, *p*<.001), high agreeableness (*r* = .15, *p*<.001) and high openness (*r* = .15, *p* = .003) were correlated with morningness at the bivariate level. Extraversion was not significantly correlated with morningness. See Table 4 for complete results of associations between personality, demographic variables, and chronotype.

**Table 4:** Bivariate Correlations Between Sleep, Personality, and Demographics.

Personality and Sleep				
	Chronotype (Higher Scores = Morningness)	Sleep Hygiene (Higher Scores = Worse)	Sleep Quality (Higher Scores = Worse)	Sleepiness (Higher Scores = More Sleepiness)
Con.	*r* = .35	*r* = −.29	*r* = −.17	*r* = −.25
	*p*<.001	*p*<.001	*p*<.001	*p*<.001
	*N* = 430	*N* = 429	*N* = 430	*N* = 431
Neur.	*r* = −.18	*r* = .37	*r* = .40	*r* = .23
	*p*<.001	*p*<.001	*p*<.001	*p*<.001
	*N* = 431	*N* = 429	*N* = 430	*N* = 432
Agree.	*r* = .15	*r* = −.17	*r* = −.06	*r* = −.18
	*p* = .002	*p*<.001	*p* = .21	*p*<.001
	*N* = 433	*N* = 431	*N* = 432	*N* = 434
Extra.	*r* = .07	*r* = −.06	*r* = −.07	*r* = .004
	*p* = .14	*p* = .24	*p* = .17	*p* = .93
	*N* = 433	*N* = 431	*N* = 432	*N* = 434
Open.	*r* = .15	*r* = −.01	*r* = −.04	*r* = −.05
	*p* = .003	*p* = .83	*p* = .45	*p* = .29
	*N* = 431	*N* = 430	*N* = 430	*N* = 432
Demographics and Sleep				
Age	*r* = .13	*r* = −.08	*r* = .04	*r* = −.12
	*p* = .007	*p* = .11	*p* = .45	*p* = .01
	*N* = 434	*N* = 432	*N* = 433	*N* = 435
Gender (Female)	*r* = .06	*r* = .08	*r* = .18	*r* = .06
	*p* = .19	*p* = .09	*p*<.001	*p* = .20
	*N* = 434	*N* = 432	*N* = 433	*N* = 435
Generation Status	*r* = .09	*r* = −.007	*r* = −.02	*r* = −.05
	*p* = .06	*p* = .89	*p* = .68	*p* = .32
	*N* = 433	*N* = 431	*N* = 432	*N* = 434
SES	*r* = .05	*r* = −.06	*r* = −.11	*r* = .008
	*p* = .32	*p* = .21	*p* = .02	*p* = .87
	*N* = 391	*N* = 389	*N* = 391	*N* = 435

*Note*. Con = Conscientiousness; Neur = Neuroticism; Agree = Agreeableness; Extra = Extraversion; Open = Openness; SES = Subjective Socioeconomic Status.

Of age, gender, generation status, and subjective (perceived) SES, only age was significantly correlated with morningness and was thus entered (centered) as the single control variable in Model I (Age ranged from 18 to 36; *r* = .13, *p* = .007). Model I accounted for 1.5% of the variance in chronotype, *F*(1, 425) = 7.49, *p* = .007, with older participants more likely to be morning types. Model II used mean-centered age and the mean-centered PCA-derived personality components (entered as a set) to predict chronotype. Model II fit better than Model I, *F*(6, 420) = 11.34, *p*<.001, and explained 12.71% of the variance in chronotype. In this final model, participants who were high in conscientiousness and openness, and low in neuroticism were more likely to be morning types (see Table 5).

**Table 5:** Multiple Regressions of Habitual Sleep Behaviors and PCA-Derived Personality Components.

Parameters b (std. b, *p*)	Chronotype		Sleep Hygiene
	Model I	Model II	Model I
Intercept	45.41 (0, <.001)	45.42 (0, <.0001)	35.52 (0, <.001)
*Age*	0.75 (–, .007)	0.45 (–, .08)	N/A
*Conscientiousness*		2.63 (.31, <.001)	−1.50 (−.24, <.001)
*Neuroticism*		−1.03 (−.12, .008)	2.10 (.34, <.001)
*Agreeableness*		0.60 (.07, .12)	−0.57 (−.09, .04)
*Extraversion*		−0.02 (−.003, .95)	0.27 (.04, .32)
*Openness*		0.84 (.10, .03)	0.17 (.03, .54)
Model Fit	*F*(1, 425) = 7.49, *p* = .007	*F*(6, 420) = 11.34, *p*<.001	*F*(5, 421) = 18.98, *p*<.001
Adjusted *R* ^2^	.015	.1271	.1743

### Sleep Hygiene

Low conscientiousness (*r* = −.29, *p*<.001), high neuroticism (*r* = .37, *p*<.001), and low agreeableness (*r* = −.17, *p*<.001) were correlated with poor sleep hygiene at the bivariate level. Extraversion and openness were not significantly associated with sleep hygiene. See Table 4 for complete results of the correlations between personality, demographic variables, and sleep hygiene.

Neither age, gender, generation status, nor subjective SES was significantly correlated with sleep hygiene at the bivariate level, so they were not used as controls. Thus, Model I used the mean-centered PCA personality factors (entered as a set) to predict sleep hygiene. Model I explained 17.43% of the variance in sleep hygiene, *F*(5, 421) = 18.98, *p*<.001, with people low in conscientiousness and agreeableness and high in neuroticism more likely to have poor sleep hygiene (see Table 5).

### Sleep Quality

Low conscientiousness (*r* = −.17, *p*<.001) and high neuroticism (*r* = .40, *p*<.001) were correlated with poor sleep quality at the bivariate level. Extraversion, agreeableness, and openness were not significantly correlated with sleep quality. See Table 4 for complete results of the correlations between personality, demographic variables, and sleep quality. For a summary of the associations between specific sleep quality domains and personality, see Table 6.

**Table 6:** Correlations between Sleep Quality Domains (PSQI) and Personality.

Sleep Domain	Con.	Neur.	Agree.	Extra.	Open.
Subjective Sleep Quality	*r* = −.09	*r* = .32	*r* = −.07	*r* = −.05	*r* = −.03
	*p* = .05	*p*<.001	*p* = .14	*p* = .32	*p* = .52
	*N* = 432	*N* = 433	*N* = 435	*N* = 435	*N* = 433
Sleep Latency	*r* = −.08	*r* = .25	*r* = −.01	*r* = −.06	*r* = −.03
	*p* = .12	*p*<.001	*p* = .79	*p* = .19	*p* = .52
	*N* = 432	*N* = 433	*N* = 435	*N* = 435	*N* = 433
Sleep Duration	*r* = −.03	*r* = .18	*r* = .01	*r* = −.04	*r* = −.02
	*p* = .56	*p*<.001	*p* = .84	*p* = .42	*p* = .69
	*N* = 432	*N* = 433	*N* = 435	*N* = 435	*N* = 433
Sleep Efficiency	*r* = −.13	*r* = .12	*r* = −.02	*r* = .01	*r* = −.07
	*p* = .006	*p* = .01	*p* = .68	*p* = .78	*p* = .15
	*N* = 432	*N* = 433	*N* = 435	*N* = 435	*N* = 433
Sleep Disturbance	*r* = −.04	*r* = .25	*r* = −.003	*r* = −.07	*r* = .02
	*p* = .36	*p*<.001	*p* = .95	*p* = .14	*p* = .69
	*N* = 430	*N* = 430	*N* = 432	*N* = 432	*N* = 430
Medication Use	*r* = −.006	*r* = .05	*r* = −.07	*r* = .07	*r* = .04
	*p* = .90	*p* = .34	*p* = .15	*p* = .14	*p* = .42
	*N* = 432	*N* = 433	*N* = 435	*N* = 435	*N* = 433
Daytime Dysfunction	*r* = −.24	*r* = .36	*r* = −.08	*r* = −.10	*r* = .0007
	*p*<.001	*p*<.001	*p* = .10	*p* = .03	*p* = .99
	*N* = 432	*N* = 433	*N* = 435	*N* = 435	*N* = 433

*Note*. Con = Conscientiousness; Neur = Neuroticism; Agree = Agreeableness; Extra = Extraversion; Open = Openness.

Of age, gender, generation status, and subjective SES, only gender and subjective SES were significantly correlated with sleep quality and were thus retained as the control variables in Model I (SES was entered centered). Note that there is a reduced *N* in the regressions for sleep quality, since subjective SES was added mid-study and thus fewer participants had complete data for this set of analyses. Model I explained 4.17% of the variance in sleep quality, *F*(2, 383) = 9.39, *p*<.001, with females more likely to have poor sleep quality. Model II used gender, mean-centered SES, and the mean-centered PCA personality factors (entered as a set) to predict sleep quality. Model II fit better than Model I, *F*(7, 378) = 13.78, *p*<.001, and explained 18.86% of the variance in sleep quality. Participants low in conscientiousness and high in neuroticism were more likely to have poor sleep quality (see Table 7).

**Table 7:** Multiple Regressions of Subjective Sleep Experience and PCA-Derived Personality Components.

Parameters b (std. b, *p*)	Sleep Quality		Sleepiness	
	Model I	Model II	Model I	Model II
Intercept	4.61 (0, <.001)	4.92 (0, <.001)	7.53 (0, <.001)	7.52 (0, <.001)
*Age*			−0.25 (–, .02)	−0.19 (–, .06)
*Gender*	1.00 (–, <.001)	0.35 (–, .19)		
*SES*	−0.14 (−0.09, .06)	−0.12 (−0.08, .08)		
*Conscientiousness*		−0.32 (−0.12, .009)		−0.66 (−0.20, <.001)
*Neuroticism*		1.05 (0.39, <.001)		0.68 (0.21, <.001)
*Agreeableness*		0.04 (0.01, .75)		−0.40 (−0.12, .008)
*Extraversion*		−0.008 (−0.003, .95)		0.29 (0.09, .06)
*Openness*		−0.01 (−0.005, .91)		−0.08 (−0.03, .58)
Model Fit	*F*(2, 383) = 9.39	*F*(7, 378) = 13.78	*F*(1, 426) = 5.86	*F*(6, 421) = 9.56
	*p*<.001	*p*<.001	*p* = .016	*p*<.001
Adjusted *R* ^2^	.0417	.1886	.0112	.1074

### Sleepiness

Low conscientiousness (*r* = −.25, *p*<.001), high neuroticism (*r* = .23, *p*<.001), and low agreeableness (*r* = −.18, *p*<.001) were correlated with high sleepiness at the bivariate level. Extraversion and openness were not significantly associated with sleepiness. See Table 4 for complete results of the correlations between personality, demographic variables, and sleepiness.

Of age, gender, generation status, and subjective SES, only age was significantly correlated with sleepiness, so it was entered as the single control variable in Model I (age was entered centered). Model I explained 1.12% of the variance in sleepiness, *F*(1, 426) = 5.86, *p* = .016, with younger participants reporting increased sleepiness. Model II used mean-centered age and the mean–centered PCA personality factors (entered as a set) to predict sleepiness. Model II fit better than Model I, *F*(6, 421) = 9.56, *p*<.001, and explained 10.74% of the variance in sleepiness. Participants low in conscientiousness and agreeableness and higher in neuroticism reported increased sleepiness. Also note that high extraversion was near standard significance (*p* = .06; see Table 7).

### Supplementary Analyses

To determine the robustness of the findings, we re-ran analyses using mean-centered personality traits. For chronotype, the parameter estimates for neuroticism and openness were in the same direction but became non-significant. For sleep hygiene, the parameter estimate for agreeableness was in the same direction but became non-significant; the parameter estimate for extraversion was in the same direction but became significant. For sleep quality, the parameter estimate for conscientiousness was in the same direction but became non-significant. For sleepiness, the parameter estimate for agreeableness was in the same direction but became non-significant, whereas the parameter estimate for extraversion was in the same direction but became statistically significant. We also tested for an interaction between PCA-derived conscientiousness and neuroticism but it was not significant for chronotype, sleep hygiene, sleepiness, or sleep quality.

## Discussion

This study is the first to comprehensively examine links between personality and sleep, including chronotype, sleep hygiene, sleep quality, and sleepiness, in a single study. Both personality and sleep are known predictors of health and longevity, and understanding their possible inter-relationships is highly informative for constructing conceptual models of long-term pathways to health. Further, the PCA-derived regression analysis technique used in this study is novel in research on sleep and personality and may be more precise in revealing core associations deserving of further research, as the unique variance of each personality factor can be distilled. Although these data do not allow us to make causal conclusions, our regression findings show that among the Big Five personality traits, conscientiousness and neuroticism are the most important correlates of sleep health. Our measures of sleep health can be divided into two domains: habitual sleep behaviors (chronotype, sleep hygiene) and subjective sleep experiences (sleep quality, sleepiness). We discuss these further below.

### Habitual Sleep Behaviors

#### Chronotype

Our study confirms the findings of much of the previous research on sleep and personality, which has focused on chronotype. Chronotype is the relatively stable preference for activity in the morning, middle of the day, or evening, and is associated with bed/wake times, body temperature, and melatonin and cortisol levels [39], [46], [47]. At the bivariate level, morning types tended to be older, more conscientious, more agreeable, more open, and less neurotic. These bivariate results are consistent with previous research, which has shown larger associations between chronotype, conscientiousness, and neuroticism (e.g., [29]) and smaller associations with agreeableness [28] and openness [48]. Using multiple linear regressions, we explained approximately 13% of the variance in chronotype. Our results suggest that conscientiousness is the best predictor of chronotype, even after controlling for age (a significant predictor of chronotype). This replicates the results of Tonetti and colleagues [29], who, using ANCOVA, found that conscientiousness best discriminated between chronotype dimensions.

#### Sleep Hygiene

Sleep hygiene involves practicing sleep-promoting behaviors and avoiding sleep-inhibiting behaviors. [42]. People with poor sleep hygiene tended to be low in conscientiousness and agreeableness and high in neuroticism at the bivariate level. Using multiple regressions, we found that high neuroticism, low conscientiousness, and low agreeableness explained approximately 17% of the variance in poor sleep hygiene, suggesting that people low in sleep hygiene may have poor emotion regulation skills, lower self-control, have difficulty getting along with others, and may be distrustful. Their poor emotion regulation skills may be interfering with their sleep-promoting health behaviors. Conversely, poor sleep hygiene may interfere with emotion regulation. This is a promising area for future longitudinal and experimental research.

This is the first study to comprehensively report associations between sleep hygiene and the Big Five personality traits. Despite the lack of research on sleep hygiene specifically, results agree with much of the previous research on personality and health behaviors more broadly. In previous research, conscientiousness has been associated with fewer risky health behaviors and more health-promoting behaviors, including lower levels of alcohol abuse, drug use, unhealthy eating, risky driving, risky sex, suicide, tobacco use, and violence [8]. Neuroticism is known to be associated with more risky health behaviors, including smoking, alcohol and drug use, and unprotected sex [10]. Thus, the findings of the current study are in line with much of the previous research on personality and health behaviors but extend it to suggest that personality also predicts sleep-promoting health behaviors. Taken together with chronotype, these data suggest that people high in conscientiousness and low in neuroticism are better able to maintain habits associated with healthy sleep.

### Subjective Sleep Experiences

#### Sleep Quality

Sleep quality, as measured by the Pittsburgh Sleep Quality Index [22], sums scores in seven components of sleep quality (subjective sleep quality, sleep latency, sleep duration, habitual sleepiness, sleep disturbances, use of sleeping medication, and daytime dysfunction) to assess global sleep quality. At the bivariate level, participants with poor sleep quality tended to be female, low in subjective SES, low in conscientiousness, and high in neuroticism. Using multiple regressions, high neuroticism and low conscientiousness explained approximately 19% of the variance in sleep quality.

Our findings are consistent with much of the previous research on sleep quality. Many studies have reported that female participants have poorer sleep quality (e.g., [49], [50]). However, other research suggests that gender differences in sleep quality may be due to gender differences in anxiety and depression [51]. Similar to the above results on age and chronotype, these results show that once personality is taken into account, gender no longer significantly predicts sleep quality. These findings may be because neuroticism, which significantly predicts sleep quality, depression, and anxiety [51], was higher in the women in our sample. Furthermore, E.M. Friedman et al. [52] found that including neuroticism attenuates associations between SES and sleep quality. Our results add to this growing body of literature by showing that the inclusion of Big Five personality factors (which may be correlated with gender and subjective SES) reduces associations between demographic factors and sleep quality to nonsignificance.

Our results confirm and extend previous research on the Big Five personality traits and sleep quality. For example, Gray and Watson [30] found that while neuroticism, low extraversion, and low conscientiousness predicted subjective sleep quality, neuroticism was the strongest predictor. Similarly, Williams and Moroz [31] found that high neuroticism and low conscientiousness were associated with poor subjective sleep quality at the bivariate level, with neuroticism the only significant predictor in multiple regressions. It is also remarkable that our results replicate work by Calkins and colleagues [53] who found that roughly 16% of the variance in sleep quality is explained by neuroticism and dysfunctional beliefs about sleep. Perhaps people low in conscientiousness and high in neuroticism may have poor sleep quality because both factors are associated with difficulty regulating emotions (anxiety, depression) and behavior (e.g., sleep hygiene). On the other hand, Dorsey & Bootzin [54] found that people high in neuroticism were likely to complain of insomnia on self-report measures, even though polysomnographic measures indicated that they did not have impaired sleep quality. Thus, similar to other health concerns [14], people high in neuroticism might also report poor sleep quality because they are especially sensitive to or worry about small deficits in sleep quality.

#### Sleepiness

Trait daytime sleepiness is a significant public health risk and is associated with sleep disorders [43]. At the bivariate level, our results show that people high in sleepiness are younger, less conscientious and agreeable, and more neurotic. Using multiple regressions, we find that low conscientiousness, low agreeableness, and high neuroticism explained approximately 11% of the variance in daytime sleepiness levels (extraversion was trending significance). These results suggest that dependable, emotionally stable, sociable people tend to be less sleepy.

Our results regarding neuroticism, extraversion, and trait sleepiness confirm previous research that used state indicators of sleepiness. However, our findings regarding conscientiousness and agreeableness are novel. Blagrove and Akehurst [55] and Mastin and colleagues [32] found that participants high in neuroticism were more affected by experimentally-induced sleep deprivation compared to individuals low in neuroticism (participants low in extraversion may also be affected [55]). Although previous research has not shown associations with conscientiousness and agreeableness, this may be because previous research used clinical samples or experimentally-induced sleep deprivation, did not report on all Big Five personality traits, or has primarily focused on associations between personality and *state* sleepiness rather than trait sleepiness. This is the first study in which personality and trait sleepiness have been examined in a non-clinical, non-laboratory setting. Future research should confirm these associations in other ecologically valid samples and examine the reasons why conscientious, emotionally stable, and agreeable people are less sleepy.

### Personality and Healthy Sleep: Dynamic Lifespan Processes

**Figure 2:**
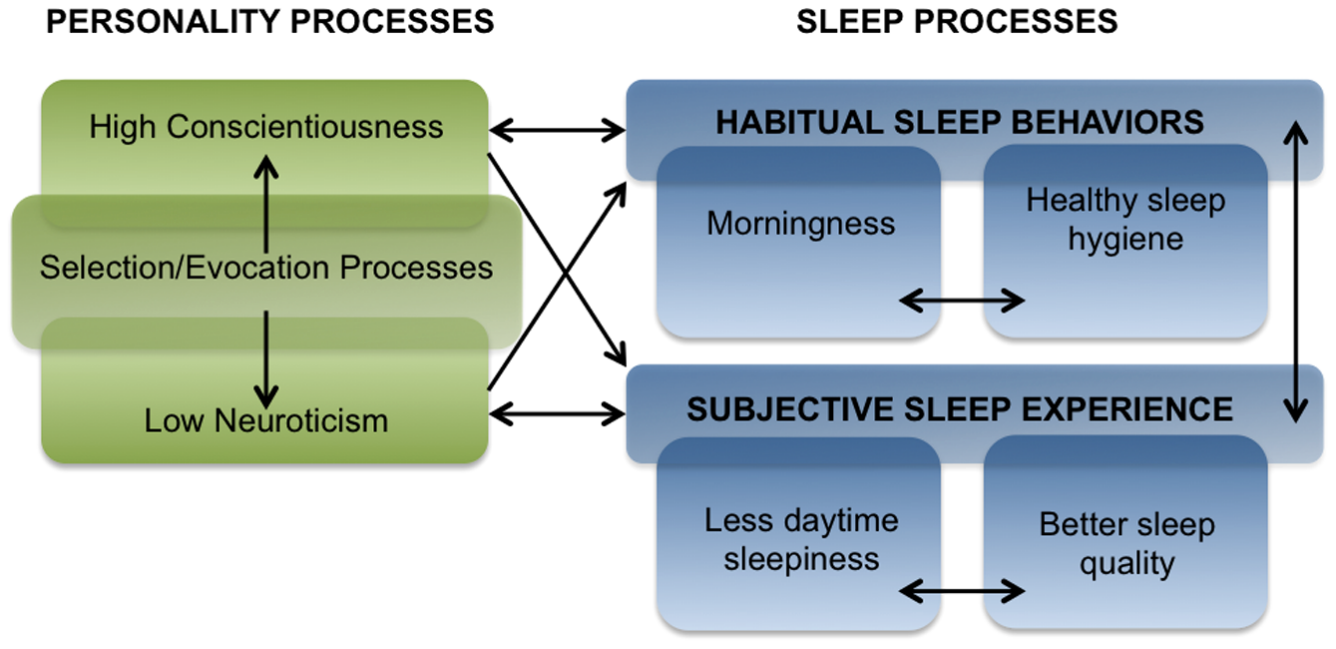
A hypothetical model of associations between conscientiousness, neuroticism, habitual sleep behaviors, and subjective sleep experience. *Note*. Additional factors may be involved. Associations likely co-occur over time.

The results of this study represent a novel exploration into associations between personality and sleep, using comprehensive, well-validated assessments in a diverse college student population. Importantly, these results can be interpreted in the context of habitual sleep behaviors and subjective sleep experiences (see Table 8 for a summary and Figure 2 for a hypothetical model). Although the correlational nature of the current study does not allow causal conclusions, we hypothesize that habitual sleep behaviors, such as usual bedtime and waketime (chronotype) as well as bedtime routines (sleep hygiene), are likely linked with personality and subjective sleep experiences (sleep quality, sleepiness) through common biological bases and complex bidirectional feedback loops. In other words, these are dynamic processes, and sleep and personality likely influence and change each other over time. This area is ripe for future research.

**Table 8:** A Summary of the Current Results.

	Chronotype (Morningness)	Poor sleep Hygiene	Poor Sleep Quality	Increased Sleepiness
Variance Explained	13%	17%	19%	11%
Conscientiousness	+	−	−	−
Neuroticism	−	+	+	+
Agreeableness		−		−
Extraversion				
Openness	+			

#### Direct Links (Including Possible Bi-Directional Links) Between Personality and Sleep

Conscientious individuals are responsible, dependable, and motivated, and may be driven to wake up earlier to accomplish more throughout the day. Individuals who have higher levels of self-control (conscientiousness) may be able to regulate their impulses better, avoid caffeine and sleep-impairing behaviors, get a good night's sleep, and avoid high levels of daytime sleepiness. Similarly, people with healthy sleep habits and positive subjective sleep experiences may have a larger pool of psychological resources (e.g., less stress, better coping abilities) that may help them develop self-control and leave them less likely to disengage from complex or long-term tasks.

Our results also show that neuroticism is associated with poor sleep. Neurotic individuals tend to experience high levels of stress [13] and have difficulties with emotion regulation [56]. Poor emotion regulation may be driven by increased negative affect (neuroticism) and poor effortful control (low conscientiousness [31]), and which are associated with anxiety and increased emotionality before bed. People with poor sleep behaviors and subjective sleep experiences may feel tired and moody throughout the day, which could promote neuroticism. Similarly, neurotic individuals might compensate for their poor nighttime sleep and high levels of daytime sleepiness by coping with caffeine, alcohol, or other behaviors that interfere with their sleep, decreasing sleep hygiene and perpetuating this destructive cycle.

#### Direct Links Between Habitual Sleep Behaviors and Subjective Sleep Experience

In addition to direct links between personality and sleep, it is important to note that sleep habits (e.g., chronotype and sleep hygiene) may feed into subjective sleep experience (e.g, daytime sleepiness and sleep quality) over time (Figure 2, blue boxes). The social and biological clocks of morning types are more likely to be aligned, and thus, these individuals may experience less social jetlag, a greater misalignment of biological and social time [57]. Morning types have better bedtime routines (sleep hygiene) and thus are able to get to sleep earlier, or the opposite may be true: morning types may experience less social jetlag, be better able to cope with the day's challenges, which may increase their sleep hygiene (i.e., they fall asleep earlier). These sleep habits then feed into subjective sleep experience: individuals with better sleep hygiene may fall asleep sooner and have more restful sleep, which would increase sleep quality and decrease levels of daytime sleepiness. Because individuals with lower levels of daytime sleepiness may avoid caffeine and other compensatory daytime behaviors that interfere with sleep, they would have better sleep hygiene, thus feeding back into these reciprocal processes over time. This is a promising topic for future research.

#### Situation Selection and Evocation

Developmental and personality theories of continuity and change throughout the lifespan suggest that individual traits are reinforced through complex processes of selection (sometimes called cumulative continuity) and evocation (sometimes called interactional continuity [58], [59]; Figure 2, green boxes). Situation selection is an active process by which the individual creates, seeks, attends to, and learns from aspects of the environment that are consistent with his or her personality traits [58], [60]. Situation evocation is a process by which people respond to the individual in ways consistent with his/her genotype [58]. Although personality is typically thought of as relatively stable and enduring patterns of thought, feelings, and behavior, it can change over time. During college and throughout adulthood, specific conscientiousness facets clearly increase, including impulse control and reliability [61], and these changes may be due to the situations individuals self-select into. Similarly, measures of trait sleepiness may also be reinforced across time. For example, individuals high in conscientiousness and low in neuroticism may be more likely to attend college and get better jobs, which would in turn serve as platforms that could further increase levels of conscientiousness and neuroticism. Evening sleep types may self-select into later classes and put themselves in situations that compromise sleep hygiene and sleep quality (e.g., consuming caffeine to compensate for social jetlag, which would thus increase sleepiness the following day. Finally, healthy sleep habits and positive subjective sleep experiences may reinforce behaviors indicative of high conscientiousness and low neuroticism, such as emotion regulation skills and self-control. Therefore, the personality traits and sleep habits develop together via selection and evocation processes.

#### Biological Bases

Although not explicitly included in our model, prior research indicates that common genetic or biological factors, such as serotonergic functioning may link personality and sleep. Serotonin acts as a general inhibitor of behavioral reactivity [62], helps people pursue goals, and may protect against psychopathology [63]. Serotonin may drive sleep behavior through two dual-processes: via a lower-order system that responds in-the-moment, and a higher-order system that responds planfully and reflectively [63]. Individuals with poor serotonergic functioning may have an enhanced lower-order system and a weaker higher-order system, which leads to impulsive behaviors and a lack of self-regulation. Importantly, serotonin has definite genetic links with conscientiousness, impulse control, and morningness [64], and some studies have linked serotonin with anxiety and neuroticism [65]. Future studies might profitably examine genetic or biological indicators of serotonin, sleep, and personality across time.

## Conclusions

In summary, this study is the first to comprehensively examine associations between the Big Five personality traits and chronotype, sleep hygiene, sleep quality, and sleepiness in a diverse college sample. Results showed that conscientiousness and neuroticism were the best predictors of sleep patterns. We summarize the results of the current study in the context of a conceptual model that bridges theories of personality development with sleep behavior. Although many open questions remain, this framework makes specific, testable predictions that can be examined in future research. Future studies examining the longitudinal associations between these factors might seek to improve sleep by intervening on specific dysfunctional attitudes, coping styles, and behaviors associated with poor sleep. Our findings suggest that future research may profitably examine the joint actions of personality and sleep in models of health and well-being.
